# Disruption of doubly uniparental inheritance of mitochondrial DNA associated with hybridization area of European *Mytilus edulis* and *Mytilus trossulus* in Norway

**DOI:** 10.1007/s00227-017-3235-5

**Published:** 2017-10-06

**Authors:** Beata Śmietanka, Artur Burzyński

**Affiliations:** Department of Genetics and Marine Biotechnology, Institute of Oceanology Polish Academy of Sciences, Powstańców Warszawy 55, 81-712 Sopot, Poland

## Abstract

Doubly uniparental inheritance of mitochondria (DUI) is best known in the blue mussel *Mytilus*. Under this model, two types of mitochondrial DNA exist: female type (F), transmitted from females to offspring of both genders, and male type (M), transmitted exclusively from males to sons. The mitogenomes are usually highly divergent, but an occasional replacement of a typical M genome by a particular F genome has been postulated to explain reduction of this divergence. Disruption of the DUI model has been reported in hybridization areas. Here, we present a new case of DUI disruption in a hybrid *M. trossulus/M. edulis* population from the North Sea (Norway). No M haplotypes derived from *M. trossulus* were identified in this population. Typical M haplotypes derived from *M. edulis* (ME) were rare. Two F-type haplogroups were found: one derived from *M. edulis* (FE) and the second derived from *M. trossulus* (FT). Many haplotypes from the FT group were recombinants, with the male CR sequence coming from the *M. trossulus* genome (FT1 haplogroup) in contrast to *M. edulis* CR as in the Baltic. FT1 haplotypes were abundant in the studied population, including homoplasmic females. However, males significantly more often carried these haplotypes; therefore, male heteroplasmy involved the original FE and recombinant FT, indicating that the FT genome undergoes masculinization. Structural similarity of FT1 CR with previously reported, masculinized Baltic haplotypes, which were derived from FE/ME recombination, provides further evidence that CR M–F recombination is a prerequisite for masculinization, also in the context of native *M. trossulus* mtDNA.

**Electronic supplementary material:**

The online version of this article (doi:10.1007/s00227-017-3235-5) contains supplementary material, which is available to authorized users.

## Introduction

Blue mussels of the *Mytilus edulis* species complex, *M. edulis*, *M. galloprovincialis* and *M. trossulus*, are widely distributed in boreal and temperate waters in the Southern and Northern hemispheres (Hilbish et al. 2000; Gerard et al. 2008). The three species hybridize in the areas of sympatry, providing a unique model to study complex patterns of genetic introgression (Gosling 1992; Riginos and Cunningham 2005). The best studied hybrid zones between *M. trossulus* and *M. edulis* are the Canadian Maritime hybrid zone, which covers a large geographical area and is known for a mosaic structure with limited gene introgression, and the Baltic Sea hybrid zone where pervasive asymmetric gene flow has led to the formation of an *M. trossulus*-like hybrid swarm. Recently, a new hybrid zone between *M. edulis* and *M. trossulus* was identified in Norwegian fjords (Ridgway and Nævdal 2004; Väinöla and Strelkov 2011) and Scottish lakes (Beaumont et al. 2008; Dias et al. 2008), but introgression patterns in this hybrid zone are still relatively poorly understood. Hybridization with *M. edulis* inhabiting these areas is far less documented; however, it is clear that, contrary to the Baltic Sea, at least Scottish *M. trossulus* did not lose its mitochondrial DNA (Zbawicka et al. 2010). In contrast to most animal species, where mitochondria are maternally inherited, *Mytilus* mussels have an unusual system of mitochondrial DNA (mtDNA) transmission, termed doubly uniparental inheritance (DUI) (Zouros et al. 1994a; Skibinski et al.1994a). Under DUI, females have only one, female (F) mtDNA inherited from their mothers, whereas males are heteroplasmic with an additional male (M) mtDNA inherited from their fathers. Sequence divergence between F and M genomes in *Mytilus* sp. approaches 30%. Faster evolution of the M genome was observed (Skibinski et al. 1994b; Stewart et al. 1995; Rawson and Hilbish 1995). Occasionally, the M genome is replaced by the F genome in a process called masculinization, decreasing the M/F sequence divergence (Hoeh et al. 1997).

Hybridization may impact mtDNA inheritance. It has been shown that paternal leakage of mtDNA occurs in interspecies hybrids in fruit fly and in mice (Matsuura et al. 1991; Shitara et al. 1998). In the context of DUI, the disruption of the model, defined as the occurrence of males lacking the M genome and females carrying the M genome has been shown both in laboratory crosses of *M. edulis* × *M. trossulus* (Zouros et al. 1994b; Wood et al. 2003) and in naturally hybridizing populations of these species (Rawson et al. 1996) as well as hybridizing populations of *M. galloprovincialis* and *M*. *trossulus* (Brannock et al. 2013). The extreme case of Baltic *M. trossulus* heavily introgressed with *M. edulis* alleles is perhaps the best example of atypical DUI, with complete loss of native *M. trossulus* mtDNA and relatively recent masculinization of F-like mitogenomes of *M. edulis* origin (Wenne and Skibinski 1995; Kijewski et al. 2006). Baltic *Mytilus* is also known for the presence of a great diversity of structural rearrangements in the control region (CR) of the masculinized mitogenomes (Burzyński et al. 2003, 2006; Zbawicka et al. 2014). Most if not all of them involve interlineage (M/F) recombination in the CR, which seems to be a prerequisite for successfully invading the paternal route of inheritance.

Here, we investigate the patterns of DUI in the recently discovered Norwegian *Mytilus* hybrid zone. We address the question whether the mitochondrial composition in males and females is disrupted due to hybridization and whether the F genome shows signs of masculinization.

## Materials and methods

### Sample collection, amplification and sequencing

Adult marine mussels *Mytilus* were collected from Stavanger Smart Farm, Norway (STA) in 2009 and from Puddefjord near Bergen, Norway (BER) in 2010 (Table 1). Prior to DNA extraction, mussels were kept frozen at −70 °C. All mussels from Bergen were successfully sexed by microscopic examination of the mantle tissue. In the Stavanger sample, sex determination was impossible, which prevented the analysis of heteroplasmy in the context of DUI in this sample.

**Table 1:** Sample geolocations, number of sampled individuals (*N*) and number of obtained coding region sequences from each haplogroup for use in interpopulation analyses

Sample	Abbreviation	Coordinates	*N*	FT	FE	ME	MT
Bergen, Norway, North Sea	**BER**	5º18′07″E, 60º23′56″N	54	38	29	7	0
Stavanger, Norway, North Sea	**STA**	5º45′32″E, 58º51′12″N	27	21	4	5	0
Punta Camarinal, Spain, Atlantic	CAM^a^	5º47′58″W, 36º04′48″N	48	na	18	18	na
Vigo, Spain, Atlantic	VIG^a^	8º39′16″W, 42º17′08″N	46	na	13	13	na
Bidasoa, Spain, Bay of Biscay	BID^a^	1º45′23″W, 43º22′48″N	51	na	20	20	na
Il de Re, France, Bay of Biscay	IDR^a^	1º22′37″W, 46º10′02″N	49	na	18	18	na
Westerschelde, Netherlands, North Sea	WES^a^	3º38′02″E, 51º26′31″N	55	na	26	26	na
Reykjavik, Iceland, Atlantic	ICE^a^	21º59′31″W, 64º03′53″N	45	na	16	16	na
Onega Bay, Russia, White Sea	ONE^a^	36º15′40″E, 64º03′10″N	38	na	10	10	na
Mecklenburg Bight, Germany, Baltic Sea	MEB^a^	12º07′16″E, 54º11′47″N	63	na	22	22	na
Gulf of Gdansk, Baltic Sea	GDA^a^	18º39′38″E, 54º33′18″N	53	na	3	3	na
Loch Etive, Scotland	LET^b^	5º10′29″W, 56º27′44″N	35	20	na	na	18
Nova Scotia, Canada	NSC^b^	60º59′39″W, 45º20′16″N	33	31	na	na	7
Aleutian Islands, Pacific	ALE^b^	160º30′16″W, 55º20′12″N	42	32	na	na	25
Vladivostok, Russia, Japan Sea	JSE^b^	131º58′11″E, 43º04′50″N	36	26	na	na	15
Howe Sound, Canada, Pacific	VAN^b^	123º14′42″W, 49º28′19″N	43	30	na	na	18

Samples abbreviated in bold are new, described for the first time in this publication

*N—* the number of analyzed individuals; FT, FE, ME, MT—the number of *nd2*-*co3* sequences of the F and M genomes in *M. trossulus* and *M. edulis*

^a ^Data from Śmietanka et al. (2009) for *M. edulis*

^b^ Data from Śmietanka et al. (2013) for *M. trossulus*

For DNA extraction, small pieces of the mantle tissue were taken, homogenized and extracted using the modified CTAB protocol (Hoarau et al. 2002). DNA was dissolved in sterile-filtered distilled water. Taxonomic identification of Norwegian mussels was carried out using two nuclear DNA markers: Me 15/16 (Inoue et al. 1995) and EF-bis (Bierne et al. 2003). An individual hybrid index was calculated by dividing the number of scored *M. trossulus* specific alleles of both markers by four; thus, individuals having all four alleles of *M. trossulus* origin get a score of one and individuals having no alleles of *M. trossulus* origin got a score of zero.

The mtDNA coding region comprising parts of *nd2* and *co3* sequences, *tRNA* ^*SER*^, *tRNA* ^*MET*^ and a short non-coding region was amplified using the F and M specific primers for *M. edulis* (Skibinski et al. 1994b; Quesada et al. 1996) and *M. trossulus* (Śmietanka et al. 2013), and verified by sequence comparison with four well-known reference genomes: female genome of European *M. edulis*—NC_006161 (Hoffmann et al. 1992), male genome of *M. edulis* from Westerschelde in the North Sea—KF632415 (Śmietanka et al. 2016), female and male genomes of pure Canadian *M. trossulus*—HM462080 and HM462081 (Śmietanka et al. 2010). The lengths of the amplified products were approximately 1280 base pairs (bp) for the F and 1540 bp for the M molecules. An attempt was made to amplify each of the four possible products from each individual to detect heteroplasmy (four PCRs per individual). Amplicons were purified by alkaline phosphatase and exonuclease III treatment (Werle et al. 1994) and sent for Sanger sequencing to Macrogen (Korea).

Additional PCRs were performed to characterize the structure of the CR. The general approach described previously was used (Filipowicz et al. 2008). Two pairs of back-to-back primers were used to detect repeats: one specific for the *M. edulis* CR (AB16–AB32) and the second, homologous pair specific for the *M. trossulus* CR (AB39–TRO3). Only the latter pair consistently amplified a 700 bp fragment in 23 individuals. The fragment was sequenced and, based on the obtained data, new diagnostic primers were designed (Supplementary Table 1). They were subsequently used to amplify and sequence parts of the CR from selected haplotypes. The PCR product of the primer pair AB15–BS1R was specific for the original F genome from *M. trossulus*, amplifying 3′ part of *lrrna* gene along with the 5′ part of the CR. Primer pair AB15–BS2 amplified a homologous fragment of the newly described genomes with altered CR structure. All sequences have been deposited in GenBank (accession numbers: MF995308–MF995392).

### Bioinformatic analysis

To obtain reliable sequences from raw sequencing reads, the reads were quality trimmed with *pregap4* and assembled by *gap4* from Staden Package version 1.7.0 (Staden et al. 2001). The alignment was constructed using ClustalX version 1.83 (Thompson et al. 1997) and manually trimmed to the same length to avoid spurious differences resulting from different read lengths.

The overall pattern of genetic polymorphism among individuals was determined by calculating standard diversity indices: haplotype diversity (hd), the number of segregating sites (*S*), *θ* per site, nucleotide diversity (*π*) and Tajima’s *D,* using DnaSP version 5.10.1 (Rozas et al. 2010). For verifying the relative pressure of purifying selection, nucleotide diversity in synonymous (*π*_S_) and non-synonymous (*π*_A_) sites was calculated using the modified Nei–Gojobori method with Jukes–Cantor correction for multiple substitutions in MEGA version 6 (Tamura et al. 2013). The invertebrate mtDNA genetic code after Hoffmann et al. (1992) was used to translate the protein-coding regions of the mitochondrial genomes. To infer recent demographic events, mismatch distributions were calculated in ARLEQUIN version 3.5.1.3 (Excoffier and Lischer 2010).

The relationship of both Norwegian samples with other populations was assessed at the interpopulation level, separately for each of the mtDNA lineages, in hierarchical AMOVA (analysis of molecular variance) implemented in ARLEQUIN. The data from Śmietanka et al. (2009) and Śmietanka et al. (2013) for *M. edulis* and *M. trossulus,* respectively, were used in these analyses. The statistical significance of the obtained indices (*Ф*_ST_ *, Ф*_SC_ *, Ф*_CT_) was estimated by 1000 permutations of the original data matrix following the Bonferroni adjustment (Rice 1989). To show the genetic relatedness of these populations, first Slatkin’s genetic distance was calculated with Tajima and Nei’s method in ARLEQUIN. Then, this distance measure was used to build neighbor-joining trees showing the genetic relatedness of populations in MEGA.

To assess the genetic relatedness among *Mytilus* mitochondrial haplotypes median-joining networks of haplotypes were constructed for three mitochondrial lineages in *network* software version 5 (http://www.fluxus-engineering.com) (Bandelt et al. 1995). Star contraction procedure was applied before network calculation (Forster et al. 1996). Different settings for the homoplasy level parameter *ε* were tested and *ε* = 30 was finally used. To account for differences in substitution rates, the transitions were weighted two times less than the transversions. The MP procedure was implemented for removing optional, uninformative branches (Polzin and Daneshmand 2003).

To statistically evaluate recombinant CR sequences, the following softwares were used: Geneconv (Padidam et al. 1999), MaxChi (Maynard Smith 1992), Chimaera (Posada and Crandall 2001), SiScan (Gibbs et al. 2000), Bootscan (Martin et al. 2005), and 3SEQ (Boni et al. 2007) as implemented in RDP (Martin et al. 2005). Statistical evaluation of sex × haplotype contingency tables was done by Fisher’s exact test, assuming *p* < 0.05 significance.

## Results

### Nuclear background

The analyzed samples of the Norwegian *Mytilus* population were dominated by *M. trossulus*-like individuals, with an average hybrid index of 0.88 (range 0.5–1).

### Mitochondrial haplotypes

We examined 84 homologous mitochondrial sequences from 59 individual mussels, amplified with lineage and species-specific primers. The obtained sequences belonged to three groups. *M. trossulus* F haplotypes (FT) were represented by 45 sequences with alignment length of 1.164 bp, *M. edulis* F haplotypes (FE) were represented by 28 sequences with alignment length of 1.183 bp. In case of the *M. edulis* M haplotypes (ME), we obtained only 11 sequences with an alignment length of 972 bp. No *M. trossulus* M-specific sequences were obtained. Standard diversity indices (Table 2) indicated significantly higher nucleotide polymorphism in ME than in the FE and FT groups of sequences. However, both FE and FT groups also significantly differed from each other. Much lower polymorphism was observed in the FT group than in the FE group, even though there were fewer FE sequences. There were 12 singletons among 18 unique FE haplotypes and 4 singletons among only 8 unique FT haplotypes. The most frequent FT haplotype (15 individuals) was common to both Norwegian localities. One haplotype was identified only in Bergen (12 individuals), while another haplotype was almost exclusively found in Stavanger (11 individuals) and only in one mussel from Bergen. Both FE and ME haplogroups had similar hd (93 and 98% respectively), but the total number of mutations and average nucleotide diversity in ME was three times higher than those in the FE group.

**Table 2:** Standard diversity indices in the three haplogroups of Norwegian *Mytilus* mussels, based on the *nd2*-*co3* coding sequence

Haplogroup	*N*	*S*	hd	*θ*	*π* (JC)	*D*_0_	*D*_4_
FT	45	8	0.67	0.002	0.001	−1.332	0.223
FE	28	68	0.93	0.015	0.009	−2.130*	−1.456
ME	11	113	0.98	0.044	0.031	−1.225	−1.371

*N* number of individuals, *S* number of segregating sites, *hd* haplotype diversity, *θ* theta (per site), *π* nucleotide diversity per site with Jukes and Cantor correction, *D*_0_ *, D*_4_ Tajima’s *D* values for non-degenerate and fourfold degenerate sites

* *p* < 0.05

**Fig. 1:**
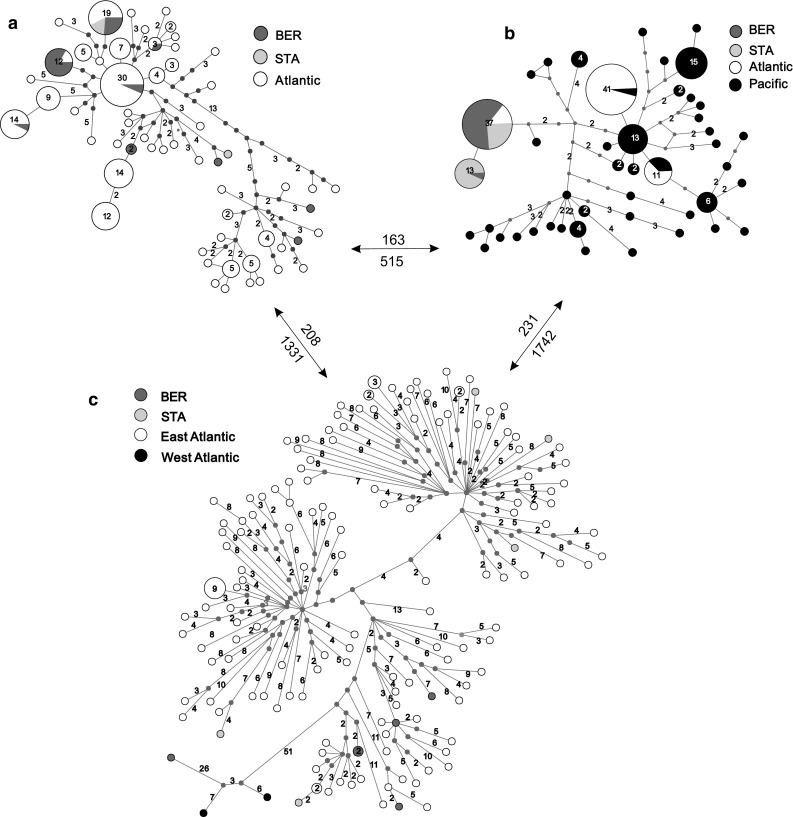
Minimum spanning networks of haplotypes for the three haplogroups found in Norwegian populations. Data for other locations are from Śmietanka et al. (2009, 2013). *M. edulis* FE (**a**), *M. trossulus* FT (**b**), *M. edulis* ME (**c**). Each circle represents either a single haplotype or a group of closely related haplotypes connected by the star contraction procedure. The circle area is proportional to the number of observed individuals carrying the specified haplotype. This number is additionally given inside the circle, except the singleton haplotypes which are not labeled. Small, gray circles represent median vectors inferred by the algorithm. Numbers on the lines connecting haplotypes indicate the number of mutational steps along each connection. Single step connections are not labeled. Numbers above the arrows between the panels indicate the mean number of base differences per sequence between the haplogroups; numbers below the arrows are the estimates after the maximum composite likelihood correction for multiple substitutions (calculated in MEGA)

Three separate MSNs generated for three mitochondrial haplogroups showed relationships of identified Norwegian haplotypes with haplotypes found in other *Mytilus* populations (Fig. 1). The FE and ME networks (Fig. 1a, c) showed spread of Norwegian sequences among other European haplotypes, except for one ME singlet which was more similar to North American M haplotypes of *M. edulis* described by Breton et al. (2006). No clade specific for the Norwegian population was visible. In the FT network (Fig. 1b), the Norwegian sequences formed two haplogroups separated by one mutational step only, with no haplotypes shared with Pacific populations. However, one of this haplogroups includes also five other Atlantic sequences from Śmietanka et al. (2013).

### Population biogeography and dynamics

**Fig. 2:**
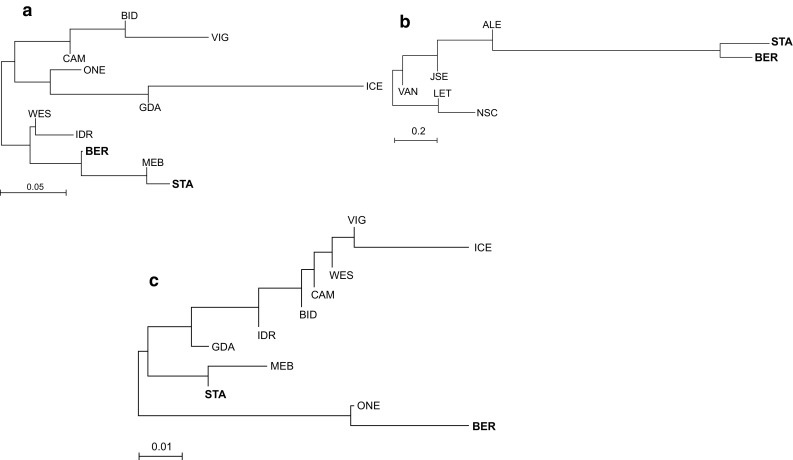
Neighbor-joining trees of *Mytilus* populations. Norwegian samples (in bold) are compared with populations described earlier (Śmietanka et al. 2009, 2013) for *M. edulis* FE genome (**a**), *M. trossulus* FT genome (**b**), *M. edulis* ME genome (**c**). The trees are based on the Slatkin’s genetic distances between populations

The neighbor-joining algorithm was used to draw interpopulation relationships based on each of the three mitogenomes separately. The trees (Fig. 2) indicated different relationship patterns between populations depending on the genome. In comparisons based on FT haplotypes (Fig. 2b), the Norwegian mussels formed the most clearly separated, distant group. The relationships suggested by the trees as well as several plausible alternative population structures for FT, FE and ME data sets were tested by AMOVA (Table 3). Consistently, the intrapopulation level (*Ф*_ST_) was the main source of diversity in all three mitochondrial genome lineages. However, only in the case of FT haplotypes, the best structure separated the Norwegian population (BER and STA) from the rest of the world with a very high between-group variation (*Ф*_CT_), at the level of 38% (4% in ME- and FE-based groupings). Notably, there was no support (*p* > 0.05) for separation of both Norwegian samples based on ME or FE data sets, regardless of the topology of the obtained trees (Fig. 2a, c).

**Table 3:** Hierarchical AMOVA in FT, FE and ME data sets

Genome	Sample grouping	*V*_a_ (%)	*Φ*_ct_	*V*_b_ (%)	*Φ*_sc_	*V*_c_ (%)	*Φ*st
FT	{VAN, JSE, ALE} {NSC, LET} {**BER**, **STA**}	38.45	0.385*	6.52	0.106*	55.03	0.449*
	{VAN, JSE, ALE} {NSC} {LET, **BER**, **STA**}	17.68	0.177	24.25	0.295*	58.07	0.419*
	{VAN, JSE, ALE} {NSC, LET, **BER**, **STA**}	9.79	0.098	31.65	0.351*	58.56	0.414*
FE	{CAM, VIG, BID} {GDA, ONE, ICE} {IDR, WES, MEB, **BER**, **STA**}	9.51	0.095*	2.95	0.033	87.54	0.125*
	{CAM, VIG, BID, GDA, ONE, ICE} {IDR, WES, MEB, **BER**, **STA**}	5.08	0.051*	6.56	0.069*	88.35	0.116*
	{CAM, VIG, BID, GDA, ONE, ICE} {IDR, WES, MEB} {**BER**, **STA**}	4.16	0.042	6.61	0.069*	89.23	0.108*
ME	{CAM, VIG, BID, WES, IDR, ICE, GDA} {MEB, **STA**} {**BER**, ONE}	6.29	0.063*	0.40	0.004	93.31	0.067*
	{CAM, VIG, BID, WES, IDR, ICE, GDA, MEB, **STA**} {**BER**, ONE}	6.49	0.065	2.14	0.023*	91.37	0.086*
	{CAM, VIG, BID, WES, IDR, ICE, GDA, MEB, ONE} {**BER**, **STA**}	3.99	0.039	2.95	0.031*	93.05	0.069*

Samples abbreviated in bold are new, described for the first time in this publication

VAN (Howe Sound, Canada), JSE (Vladivostok, Russia), ALE (Aleutian Islands), NSC (Nova Scotia, Canada), LET (Loch Etive, Scotland) from Śmietanka et al. (2013); CAM (Punta Camarinal, Spain), VIG (Vigo, Spain), BID (Bidasoa, Spain), GDA (Gulf of Gdansk, Poland), ONE (Onega Bay, Russia), ICE (Reykjavik, Iceland), IDR (Il de Re, France), WES (Westerschelde, The Netherlands), MEB (Mecklenburg Bight, Germany) from Śmietanka et al. (2009); BER (Bergen, Norway), STA (Stavanger, Norway)

FT—*M. trossulus* F genome, FE—*M. edulis* F genome, ME—*M. edulis* M genome

*V*(%)—variance components: a—among groups; b—among populations within groups; c—within populations, *Φ*_CT_, *Φ*_SC_, *Φ*st—fixation indices, * *p* < 0.01

The Tajima’s *D* statistics for fourfold degenerate as well as non-degenerate sites (Table 2) indicated an excess of low-frequency polymorphisms producing negative *D* values. However, within FE sequences the significantly (*p* < 0.05) negative *D* value was observed only in the case of non-degenerate sites, most likely because only this category of substitutions had adequate representation.

The negative Tajima’s *D* suggested expansion of the FT haplogroup. To assess the timing of this apparent event mismatch, analysis was conducted on these sequences (Supplementary Fig. 1). This analysis confirmed a past or still ongoing population expansion (raggedness index = 0.027; SDD = 0.004), with a timing defined at *τ* = 1.98.

### CR recombination

**Fig. 3:**
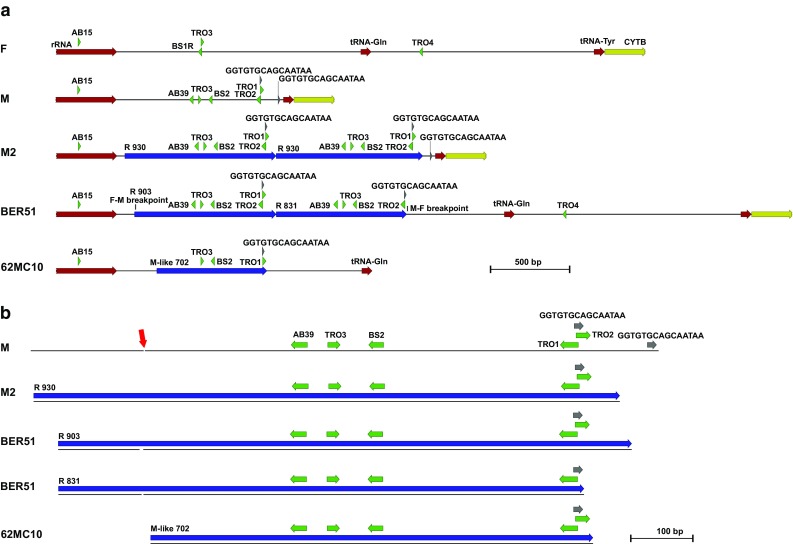
Genetic maps of mtDNA control region structure in *Mytilus trossulus* M genomes (M—HM462081 and M2—GU936627), F genome (HM462080), Baltic *Mytilus trossulus* recombinant (62MC10—KM192133) and Norwegian *Mytilus trossulus* recombinant BER51 (MF995392). The location of primers used in analysis is visualized (in green) as well as the major features: protein-coding (yellow) RNA genes (brown), repeats (blue) and other features (gray). The red arrow points at the location of the element critical for paternal transmission (details provided in Supplementary Fig. 2)

All Norwegian individuals having a coding region haplotype from the FT haplogroup were uniquely positive for the AB39-TRO3 (this primer pair amplifies the repeat part of the sequence specific for *M. trossulus* CR) as well as AB15-BS2 PCR (this primer pair amplifies the fragment of the newly described genomes with altered *M. trossulus* CR structure) products. However, some individuals were positive for the alternative, AB15-BS1R PCR product from the *lrrna*–CR boundary, identical to the homologous region of the Canadian *M. trossulus* F genome (HM462080). This indicated the presence of two different FT haplotypes: FT1 with changed CR structure and FT2 with unchanged, native F structure. The two were positively scored together in two individuals only. In the remaining FT-positive individuals only the FT1 haplotype was present. One of these haplotypes was selected for detailed analysis of the CR structure. It was possible to obtain the sequence of two repeats and the flanking sequences of the CR from this individual (Fig. 3a). Given no visible variation in the length of the PCR products obtained with back-to-back primers (AB39-TRO3) for all FT1-positive individuals, this structure is likely representative for the whole FT1 haplogroup. The CR structure of this mitogenome is complex. Comparison of its sequences with Canadian M and F *M. trossulus* reference sequences (Śmietanka et al. 2010) with the Scottish M genome of *M. trossulus* GU936627, having repeated large part of the CR (Zbawicka et al. 2010), and with the very rare recombinant Baltic *M. trossulus* mitogenome KM192133 (Zbawicka et al. 2014) was made. It suggests that FT1 genomes have an approximately 900 bp-long insertion very similar to M genome sequence from native Canadian *M. trossulus* in the CR, repeated several times in a tandem array. The positions of recombination breakpoints were confirmed by all applied methods with high confidence (*p* < 0.05). This structure is similar to the mentioned Baltic KM192133 haplotype, but the span of the M-type recombinant sequence in the FT1 haplotypes is wider, similar to the span of the repeats in the Scottish M genome (Fig. 3b).

### Doubly uniparental inheritance

Similar numbers of females (29) and males (26) were identified in the Bergen sample. Heteroplasmy was scored whenever more than one haplotype was amplified from the same individual (Table 4). It was found in more than half of males, but also in nine females, and the sex bias of heteroplasmy toward males was non-significant. Usually, heteroplasmy involved two F-type mitogenomes: one from FT and the second from the FE haplogroup. Typical M/F heteroplasmy was observed rarely, apparently because a typical M genome of *M. trossulus* was entirely missing and the typical M genome of *M. edulis* (ME) was rare. One male with three different genomes FE/FT/ME was also observed. Despite the lack of significant sex-biased heteroplasmy, FT1 haplogroup was significantly (*p* < 0.05) sex-biased: FT1 haplotypes were detected significantly more often in males.

**Table 4:** Distribution of haplogroups in sexed *Mytilus* individuals from the Norwegian population

Gender	*N*	mtDNA genome				Heteroplasmy						
		FE	FT1	FT2	ME	Total	FE/FT1	FE/FT2	FE/FT1/FT2	FE/ME	FT1/ME	FE/FT1/ME
Males	26	13	23	2	4	14	8	0	2	1	2	1
Females	29	16	17	2	3	9	4	2	0	1	2	0
Total	55	29	40	4	7	23	12	2	2	2	4	1

*N*—number of specimens; FE—*M. edulis* F genome; FT1—*M. trossulus* recombinant F genome; FT2—*M. trossulus* F genome; ME—*M. edulis* M genome

## Discussion

The pattern of mitochondrial polymorphisms expected of a DUI animal, with one haplogroup exclusively associated with males, is not present in the Norwegian *Mytilus* population (Table 4). Under undisrupted DUI, no males should be homoplasmic and no females should be heteroplasmic. However, no significant sex bias in detectable heteroplasmy was observed in the studied population. This indicates that DUI is disrupted. However, the distribution of haplotypes from the FT1 haplogroup was significantly biased toward males. The FT1 haplogroup was apparently overrepresented in the studied population, in comparison to other *M. trossulus* populations (Fig. 1b), while the typical M-type haplotypes were greatly diminished. This pattern is consistent with the notion that FT1 haplogroup is derived from a haplotype capable of switching the inheritance mode from the normal maternal to paternal transmission. The FT1 haplogroup had a relatively low genetic diversity (Table 2) and underwent an expansion with a timing (Supplementary Fig. 1) similar to the most abundant F-type *M. trossulus* clade (Śmietanka et al. 2013). Therefore, the origin of this phenomenon can be traced to a very recent, postglacial expansion of *M. trossulus* in the Atlantic (Rawson and Harper 2009; Śmietanka et al. 2013).

Previous studies have shown that parts of the CR are involved in masculinization of mitochondrial genomes in *Mytilus* (Burzyński et al. 2003, 2006). The detailed analysis of the CR structure in the *M. edulis* species complex (Rawson 2005; Cao et al. 2009) showed the complex pattern of events leading to the atypical present-day structure of the maternally inherited native mitogenomes of *M. trossulus*. The CR in this case always contains a piece of M-like DNA, despite following the maternal inheritance pattern. However, it has been recently shown that the important paternal inheritance determinants are missing in this mitogenome (Kyriakou et al. 2015). Interestingly, the FT1 haplotypes have undergone a secondary recombination and acquired a fragment of CR from a typical M mitogenome of *M. trossulus* and this fragment also includes the element described as critically important for paternal inheritance (Fig. 3b, Supplementary Fig. 2). Consequently, this new case of M–F interlineage recombination, observed in the phylogenetic context of a native *M. trossulus* mitochondrial DNA, represents in essence an independent replicate of similar events responsible for the generation of Baltic recombinant haplotypes. The Baltic situation is, however, different in two aspects: the recombining haplotypes were of *M. edulis* origin and the masculinization was complete—the DUI expectations were fully met, only the identity of the paternally transmitted genomes had changed (Burzynski et al. 2006). Nevertheless, the fact that different mitochondrial genomes undergo similar structural rearrangements which may lead to their masculinization confirms that these processes may indeed be causally linked, as postulated previously (Burzynski et al. 2003). The similarity of rearrangements in mtDNA CR between Baltic and Norwegian *M. trossulus* mussels is apparent not only because M-derived sequences were involved in both cases, but also because both structures involve the presence of tandem repeat arrays. The formation of such arrays within the CR of M-type genomes was postulated to be the first step in the proposed evolutionary scenario (Burzynski et al. 2006). This scenario conveniently explained the reversed order of M-type segments within the recombinant genomes, and the postulated M-type haplotypes with repeat structures were later identified (Filipowicz et al. 2008). In the FT1 haplotypes, the M-derived segments are not reversed, they are fully collinear with the respective parts of the native F genome and so the emergence of tandem repeat arrays could have been secondary in this case. However, a haplotype with repeat structure in the CR under native *M. trossulus* M background has already been found in Scottish mussels (Fig. 3, haplotype M2) (Zbawicka et al. 2010). The comparison of the span of the arrays found in BER51 and M2 haplotypes (Fig. 3b) suggests that the intermediate similar to M2 could have been involved in the formation of the repeat array also in this case. Notably, in the CR structure of the typical *M. trossulus* M genome, there is a 15 bp-long sequence motif “GGTGTGCAGCAATAA” repeated twice at the proximity of the 3′ end of the CR (Fig. 3). Apparently, all analyzed repeat units from BER51 and M2 end between the two copies of this motif (Fig. 3b). This is compatible with the scenario under which the motif provides the basis for repeat generation through polymerase slippage during replication, with subsequent homologous M–F recombination between repeat-bearing M genome and regular F genome.

Biogeographic inferences based on mitochondrial markers are always risky in hybridization areas, due to difficulties in distinguishing introgression from incomplete lineage sorting (Bierne et al. 2003; 2006). It gets particularly difficult in DUI species: it is easy to confuse lineage-specific events with general demographic events. Here, the FT1 haplogroup shows signs of an ongoing expansion after bottleneck and a very small effective population size. These signs may, however, be due to the expansion of FT1 haplotypes into both inheritance routes rather than due to the general demographic events concerning Norwegian *M. trossulus*. Likewise, the distinctiveness of the Norwegian *M. trossulus* visible in the FT haplotype data may not reflect the true population genetic structure, but rather the recent sweep of the mitochondrial lineages by a very young group of mitogenomes, primarily caused by their acquisition of M-type CR.

The key questions remain, of course, what promotes masculinization of these mitogenomes and what is its apparent connection with hybridization. It has been long recognized that hybridization does act it two ways on mtDNA. It increases the chance of non-standard mtDNA inheritance, such as paternal leakage (Kondo et al. 1990). It must also pose an additional selective pressure on mtDNA due to cyto-nuclear conflict in hybrid individuals. In DUI animals, the occasional switch in inheritance route may therefore be more likely in interspecies hybrids (Wood et al. 2003). However, it is unclear why the M genome is eliminated and replaced by a new F-derived one. In this context, it is interesting to note that in the Norwegian *Mytilus* hybrid zone, most individuals seemed to be highly admixed (*M. trossulus*-like). It is possible that FE/FT heteroplasmy (the most common case) is a solution for avoiding cyto-nuclear mismatch. If the original M genomes cause so much hybrid incompatibilities that they are eliminated, this would facilitate the masculinization process. Further research, involving both mitogenomic and general genomic data, is needed to verify these hypotheses.

## Electronic supplementary material

Below is the link to the electronic supplementary material.

Supplementary material 1 (PDF 52 kb)

Supplementary material 2 (PDF 48 kb)

Supplementary material 3 (PDF 158 kb)
