# Supplementary material for: Disruption of doubly uniparental inheritance of mitochondrial DNA associated with hybridization area of European Mytilus edulis and Mytilus trossulus in Norway
Source: Mar Biol. 2017 Oct 6;164(11):209. doi: 10.1007/s00227-017-3235-5 (PMC5630648; doi:10.1007/s00227-017-3235-5)
Supplement: Supplementary file 1 — Supplementary material 1 (PDF 52 kb) [file 227_2017_3235_MOESM1_ESM.pdf]

Disruption of doubly uniparental inheritance of mitochondrial DNA associated with hybridization area of European *Mytilus edulis* and *Mytilus trossulus* in Norway

Marine Biology

Beata Śmietanka, Artur Burzyński

Affiliation of authors:

Institute of Oceanology Polish Academy of Sciences, Department of Genetics and Marine Biotechnology, Powstańców Warszawy 55, 81-712 Sopot, Poland

Corresponding author: Beata Śmietanka, bsmietanka@iopan.gda.pl

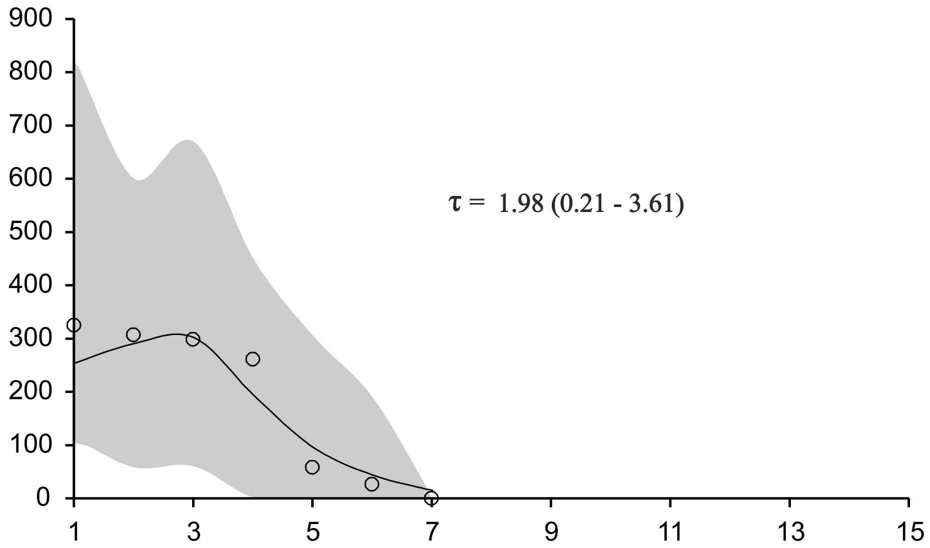

Supplementary Fig 1 Mismatch distribution of *nd2-co3* part of sequence for Norwegian *M. trossulus* F genome. The observed frequencies are presented as open circles; the line shows expected frequencies based on the best-fitted population growth-decline model.
