# Supplementary material for: Disruption of doubly uniparental inheritance of mitochondrial DNA associated with hybridization area of European Mytilus edulis and Mytilus trossulus in Norway
Source: Mar Biol. 2017 Oct 6;164(11):209. doi: 10.1007/s00227-017-3235-5 (PMC5630648; doi:10.1007/s00227-017-3235-5)
Supplement: Supplementary file 2 — Supplementary material 2 (PDF 48 kb) [file 227_2017_3235_MOESM2_ESM.pdf]

Disruption of doubly uniparental inheritance of mitochondrial DNA associated with hybridization area of European *Mytilus edulis* and *Mytilus trossulus* in Norway

Marine Biology

Beata Śmietanka, Artur Burzyński

Affiliation of authors:

Institute of Oceanology Polish Academy of Sciences, Department of Genetics and Marine Biotechnology, Powstańców Warszawy 55, 81-712 Sopot, Poland

Corresponding author: Beata Śmietanka, bsmietanka@iopan.gda.pl

```
HM462081  TGTATCTAAACCATGGGGTGTGTTTAGGGTGTGTGCCTGTAAGAAAAAAAAAAAAAAAAAAAAAAAAAAA---CCGTGAAAAGCCTTAAGAATGAGGCTTTATGGCGTACTTCATTTCCTTGCCACAGGTAGGGACAGTGACCATGACCCCTTT
FT  CR    TGTATCTAAACCATGGGGTGTGTTTAGGGTGTGTGCCTGTAACAAAAAAAAAAAGAAAAA------------CCCGTGAAAACCCTTAAGAATGAGGCTTTATGGCGTACTTCATTTCCTTGCTACAGGTAGGGGCAATGACCATGACCCCTTT
HM462080  TGT-TCTAAAA-ACGCGGCGTGTAAAGGATTTGTGCTAGTAACAGGCAAAAACAAAAAAA-------------CCGTGGAAACCCTTAAGAATGAGGTTTTATAGTGTACCGCATTTCCT-GTCACCGGCCGGTACAGTGACCGTAAGGCCTGT
                                                                            AAACACT
```

Supplementary Fig 2 The part of the M-type VD1 primary structure from two reference Canadian *M. trossulus* M and F genomes (HM462081, HM462080) and corresponding part of *M. trossulus* recombinant genome from Bergen. Presented sequence domain is suggested to be crucial in paternal transmission of mtDNA. The adenine tract (frame) and the binding site (gray box) are key for the formation of specific complex with protein factor that occurs only in male gonads (Kyriakou et al. 2015).
