# Supplementary material for: Disruption of doubly uniparental inheritance of mitochondrial DNA associated with hybridization area of European Mytilus edulis and Mytilus trossulus in Norway
Source: Mar Biol. 2017 Oct 6;164(11):209. doi: 10.1007/s00227-017-3235-5 (PMC5630648; doi:10.1007/s00227-017-3235-5)
Supplement: Supplementary file 3 — Supplementary material 3 (PDF 158 kb) [file 227_2017_3235_MOESM3_ESM.pdf]

Disruption of doubly uniparental inheritance of mitochondrial DNA associated with hybridization area of European *Mytilus edulis* and *Mytilus trossulus* in Norway

Marine Biology

Beata Śmietanka, Artur Burzyński

Affiliation of authors:

Institute of Oceanology Polish Academy of Sciences, Department of Genetics and Marine Biotechnology, Powstańców Warszawy 55, 81-712 Sopot, Poland

Corresponding author: Beata Śmietanka, bsmietanka@iopan.gda.pl

Supplementary Table 1 Primers used for sequencing part of control region in *M. trossulus* recombinant genome from Bergen sample

Name	Region	Strand	Specificity	Sequence	Reference
TRO1	CR	F	M	GTGCAGCAATAAAACGAGGGTAA	DQ013366
TRO2	CR	R	M	GCACACCACATTTTCATTAAATCTATTTA	DQ013366
TRO3	CR	F	F, M	GTAAGCATTTTTCAGAGTCA	AY823625
TRO4	CR	F	F	ATTATTTATGTCATTACAGATCC	AY823625
AB15	*rrnaL*	F	F, M	TTGCGACCTCGATGTTGG	NC_006161
AB39	CR	R	M	CAGGCTGTAAAGCATAATCTAAAACA	DQ013366
BS1R	CR	R	F	TGCTTACTCACTGTAAAGGGTTCA	HM462080
BS2	CR	R	M	AACACAGGAAGCAAATAGCTCTAT	HM462081
